# Actin dynamics in protein homeostasis

**DOI:** 10.1042/BSR20210848

**Published:** 2022-09-12

**Authors:** Thomas D. Williams, Adrien Rousseau

**Affiliations:** MRC-Protein Phosphorylation and Ubiquitylation Unit, School of Life Sciences, University of Dundee, Dow Street, Dundee, DD1 5EH, U.K.

**Keywords:** Actin, Cell stress, protein homeostasis, protein regulation, proteostasis

## Abstract

Cell homeostasis is maintained in all organisms by the constant adjustment of cell constituents and organisation to account for environmental context. Fine-tuning of the optimal balance of proteins for the conditions, or protein homeostasis, is critical to maintaining cell homeostasis. Actin, a major constituent of the cytoskeleton, forms many different structures which are acutely sensitive to the cell environment. Furthermore, actin structures interact with and are critically important for the function and regulation of multiple factors involved with mRNA and protein production and degradation, and protein regulation. Altogether, actin is a key, if often overlooked, regulator of protein homeostasis across eukaryotes. In this review, we highlight these roles and how they are altered following cell stress, from mRNA transcription to protein degradation.

## Cell and protein homeostasis

All cells are constantly subjected to a barrage of ever-changing environmental insults. Changes in nutrient status, temperature, salt and oxygen concentrations, exposure to toxic compounds, and many further insults cause cell stress [1–6]. To survive, cells must respond to these stresses by either adapting gene expression to maintain cellular homeostasis or differentiating to a more stress resistant form [7–10]. Altering the composition of the cellular proteome is key to maintaining cellular homeostasis upon stress.

**Figure 1:**
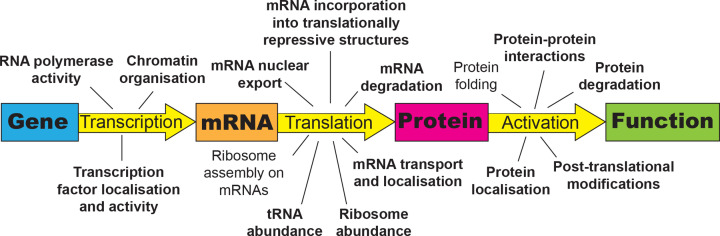
Processes contributing to protein homeostasis and the role of actin Protein homeostasis is regulated by the transcription of genes into mRNA, the translation of mRNAs into proteins, and the stability and activity status of those proteins. Aspects contributing to each of these elements of protein homeostasis are shown, with those known to have a contribution from actin structures picked out in bold.

Cells must have the optimal amounts of each protein at the correct location performing the correct function in any given environment. This is achieved by an extraordinarily complex collection of processes collectively termed protein homeostasis, or proteostasis, which involves the coordinated regulation of every step in the process of turning genes into mRNA into protein (Figure 1). Protein homeostasis is regulated at many levels. mRNA transcription, degradation and localisation all affect the availability of mRNA, and hence the potential rate of synthesis of encoded proteins. mRNA translation is highly regulated and varies depending upon the particular mRNA, with most being translated primarily in the absence of stress, while others are more translated following particular stresses [11–13]. Some mRNAs are transported to defined subcellular locations to be translated [12,14–16]. Following translation, the nascent chains must be folded into a 3D protein structure. Nascent chain folding depends on the availability of protein chaperones and the intracellular conditions [17]. Post-translational modifications such as phosphorylation, which can regulate protein activity and localisation, depend upon the activity of other proteins such as kinases and phosphatases, which are themselves subject to regulation depending on the cellular environment [18–22]. Finally, regulated degradation of proteins is critical for ensuring the levels and the biological activity of each protein are optimal [23,24].

Actin has been reported to function in many of the processes described above, making it an important regulator for protein, and therefore cell, homeostasis. These roles are discussed throughout the rest of this review.

## Actin structures within cells

The cell cytoskeleton is composed of actin filaments and microtubules in plants, while fungi additionally contain septins, and animal cells contain all three components as well as intermediate filaments [25–27]. Of the cytoskeletal components, actin structures have a notably large degree of organisational diversity, and these disparate structures can be rapidly altered to adapt to local conditions [28–30]. Individual globular (G-)actin subunits bound to ATP are assembled into straight or branched chains of filamentous (F-)actin following ATP hydrolysis by nucleation promotion factors. Actin chains are either assembled into linear structures, by formins, or into branched structures by the Arp2/3 complex [31]. Beside formins, linear actin structures can be synthesised by the co-ordinated activity of tandem W-domain nucleators (e.g. spire and cobl) which, through distinct mechanisms, can form a nucleus of F-actin, and Ena/VASP elongation factors, which can extend this nucleus into a filament [32]. The final actin structure is defined by the amount of actin filament branching, their length, and the degree and orientation of filament bundling, which depends on interactions with specific proteins, molecular crowding, and cation concentration [33,34]. These actin structures can be quickly disassembled, allowing rapid adaptation to a change in cell circumstances [28,35].

**Figure 2:**
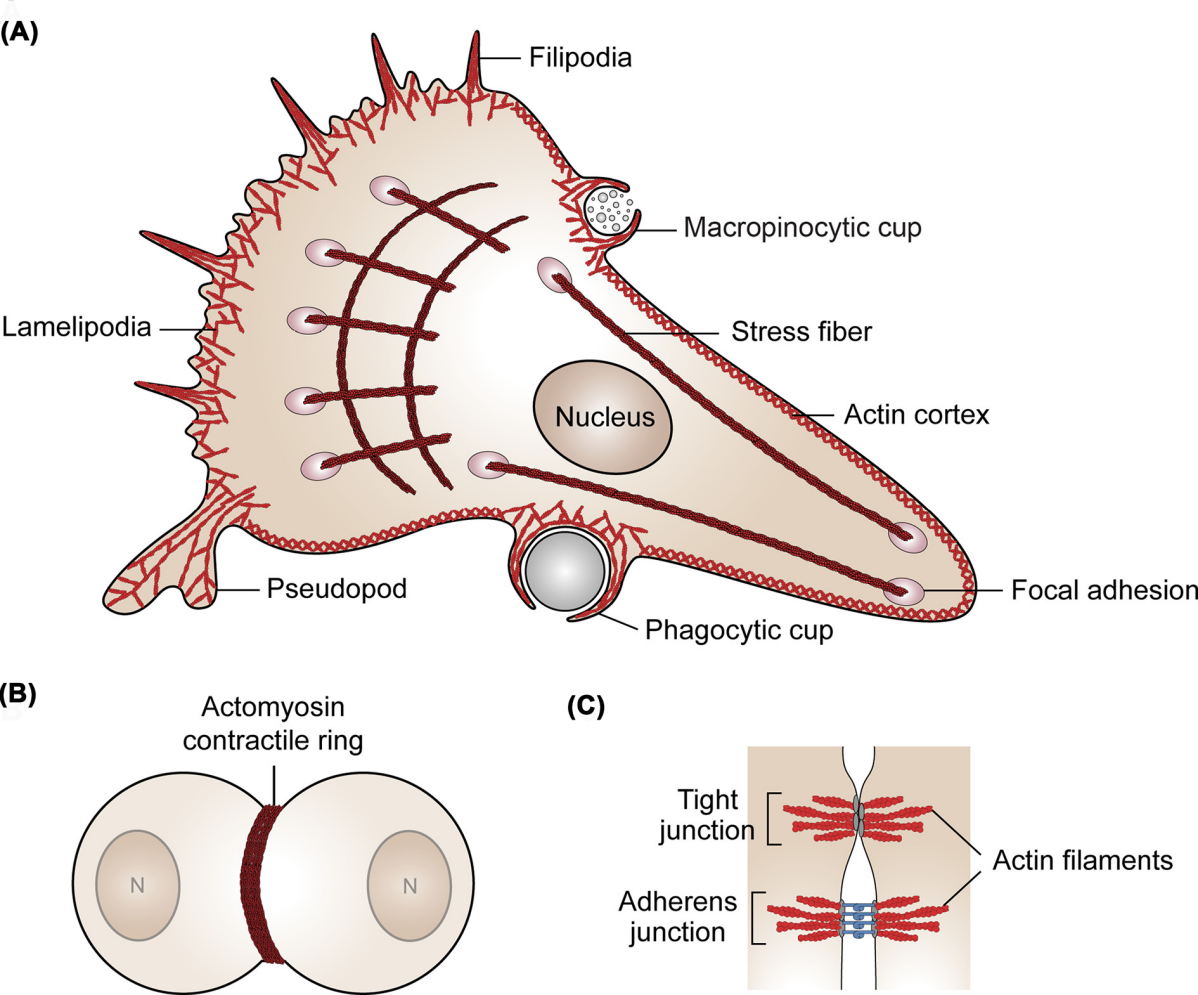
Mammalian actin structures Diverse actin structures of mammalian cells are shown. (**A**) Actin structures in motile cells. Filopodia, lammelipodia, pseudopodia, and focal adhesions all contribute to cell motility while macropinocytic and phagocytic cups take up extracellular material. Stress fibres and the actin cortex help define cell shape and stiffness. (**B**) A dividing cell showing the actomyosin contractile ring structure. (**C**) Actin structures mediate junction formation between cells.

For cells lacking a cell wall, including animal cells, actin helps define cell shape. A great variety of structures are formed, which can undergo rapid alteration (Figure 2A–C). These structures include many used for cell migration (focal adhesions, pseudopods, lamellipodia, and filopods), nutrient acquisition (membrane ruffles, macropinocytic cups, and phagocytic cups), protein and vesicle trafficking (filaments and tunnelling nanotubes), surface adhesion (focal adhesions), cell–cell contacts (tight junctions and adherens junctions), and cell division (contractile rings) [30,36–46]. Nuclear actin exists in monomeric, short polymeric, and rod forms; however, these structures remain somewhat mysterious due to poor binding by commonly used actin markers [47,48].

**Figure 3:**
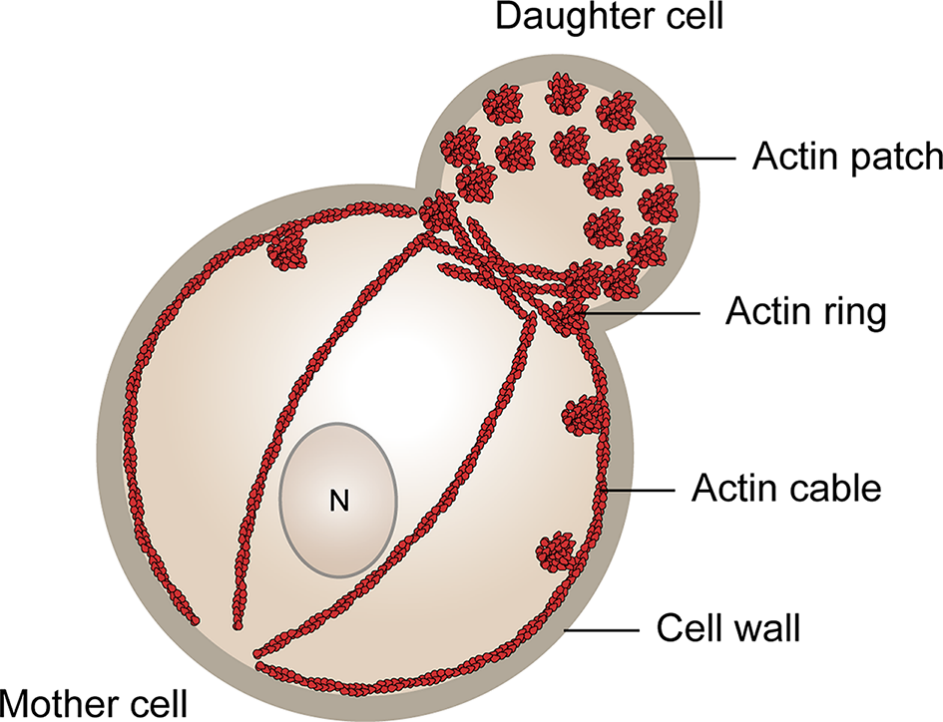
Budding yeast actin structures Budding yeast cells have a polarised distribution of actin. A high concentration of cortical actin patches (CAPs), defining sites of endocytosis, is observed in the daughter cell relative to the mother cell. Actin cables run from the daughter (bud) to the mother cell. A ring of actin defines the bud neck dividing the mother from the daughter, and later in the cell cycle forms the contractile ring.

There are fewer actin structures in cells containing a cell wall, as the manipulation of cell shape is not a prevalent function. Plant actin is predominantly in the form of filaments which are used for trafficking [25]. Budding yeast have three major actin structures [49] (Figure 3). Actin cables are formed of bundled straight chains which can stretch over the entire cell and are used for intracellular transport and establishing polarity [14,50,51]. Total disruption of the actin cables by removing the bundling tropomyosin proteins is lethal [52]. These structures are comparable to the actin filament network observed in mammalian cells, which is likewise used for intracellular transport. Cortical actin patches (CAPs) are dense meshworks of highly branched actin that enable endocytosis and have structural similarities to mammalian focal adhesions [53]. CAPs are further polarised to the bud during normal cell growth and become depolarised upon cell division and some stresses [54–58]. Finally, the actomyosin ring is important for proper cell division to occur and functions analogously to contractile rings [59]. These structures are tightly controlled by an array of actin-binding proteins (ABPs) [60].

While actin is typically thought of as one protein, it is encoded in most organisms by numerous genes which encode highly similar proteins. This is likely partly to facilitate proper actin expression and to optimise translation for different cellular conditions. It is taken to the extreme in *Dictyostelium discoideum*, an organism which constitutively undergoes rapid and extensive remodelling of its cortical actin, which has over 30 different actin-coding genes, including 17 copies of *act8*, although most of these are minimally expressed [61,62]. Mammals have six actin isoforms: three α, one β, and two γ, of which only the β and one type of γ are not restricted to pools of muscle cells. Despite the sequence similarity at the protein level, there are variations at the nucleotide level which contribute to different rates of mRNA translation and localisation [63,64]. That these differences in isoform properties is functionally important is underscored by β-actin knockout mice being embryonically lethal while γ actin is not, the importance of β-actin in neurodevelopment, and differences in cell motility when mRNA regulatory elements are swapped between different isoforms [65–69]. While it is clear that these differences are important, the function of different actin isoforms in protein homeostasis is unknown.

In addition to the commonly described functions of actin outlined in the previous paragraph, it has become clear that the dynamic regulation of actin has a major role in maintaining protein homeostasis. Actin structures are involved at nearly every stage of protein regulation, as well as functioning as environmental stress sensors. This review will focus on budding yeast and animal cells, as the most investigated systems for the role of actin in maintaining protein homeostasis.

## Actin responses to environmental change

Several stresses alter cellular actin structures in both yeast and mammalian cells. In yeast, actin becomes specifically depolarised following multiple stresses, such as cell membrane, nutrient, and osmotic stresses, with actin cables becoming less prominent and actin patches redistributing from predominantly in the bud to being observed throughout bud and mother cell [54–57]. Actin has therefore been suggested to be a stress biosensor in yeast, and its depolarisation may act as an ‘emergency brake’ to prevent cell growth under adverse conditions [28]. As cells adapt to their changed environment, the actin cytoskeleton is repolarised and growth resumes.

In mammalian cells, actin structures are highly responsive to environmental change. Actin rods form in the nucleus following oxidative stress, certain bacterial toxins can either promote or inhibit actin polymerisation, which can affect focal adhesion and stress fibre organisation, while heat shock affects actin filaments [70–73]. Conversely, upon growth factor stimulation, cell actin dynamics are modified to increase motility and large actin-driven membrane ruffles are formed, which can circularise and close to form macropinosomes [74–76]. Macropinosomes have been postulated to stimulate signalling of the growth promoting kinase complex mTORC1, in addition to providing extracellular nutrients, providing a link between the induced actin dynamics and enhanced anabolic processes [77].

While we are some way off a complete description of actin responses to changing environments, it is clear that actin is highly responsive to environmental change, and this responsiveness is important for cell adaptation to altered environments.

## Actin in RNA synthesis

DNA transcription into mRNA is regulated by chromatin structure, transcription factor function, promoter activity and RNA polymerase activity and function. Nuclear actin, the structures of which remain unclear, but which can be monomeric, in short polymers, or in rods, affects each of these processes [47,48]. The proportions and levels of each nuclear actin structure are likely to be important for regulating transcription. While studying nuclear actin is challenging, fascinating insights into its function have been gleaned through specific removal and addition of nuclear actin structures using protein depletion and microinjection techniques.

The regulation of chromatin structure by nuclear actin controls the accessibility of genes for transcription. Several chromatin modifying complexes contain monomeric actin, which plays a key role in the modulation of chromatin dynamics and structure in both yeast and humans [78]. Chromatin remodelling by actin has been actively implicated in cellular reprogramming, where the cells’ transcriptional output changes significantly. Actin mediated recruitment of chromatin remodelling components allows induction of pluripotency in *Xenopus laevis* [79]. Depleting nuclear actin leads to decreased expression of the differentiation factor CEBPA during adipogenesis in MEFs, resulting in decreased chromatin accessibility and impairing adipocyte differentiation [80].

Transcription factor abundance and function are regulated, in many cases, by nuclear actin. Overexpression of actin targeted to the nucleus by the addition of a nuclear localisation sequence increases transcription of many transcription factors in *Xenopus* oocytes, which will affect expression of target genes [81]. Actin also associates directly with some transcription factors, such as MRTF-A, which is involved in differentiation and capillary formation [82,83]. MRTF-A is retained in the cytoplasm when associated with cytoplasmic G-actin, but following serum stimulation, a burst of actin polymerisation occurs reducing the concentration of G-actin, thereby inducing MRTF-A nuclear translocation [84]. Similarly, nuclear actin interacts with estrogen receptor α following its activation and nuclear translocation. In the nucleus, estrogen receptor α functions as a transcriptional regulator to promote proliferation and motility, although the significance of its interaction with actin remains unclear [85–87].

Actin is essential for activity of RNA polymerases I, II, and III in mammalian cells, associating with each [88–93]. RNA polymerases I and III primarily produce rRNA, tRNA and other non-coding RNAs, while RNA polymerase II synthesises mRNA and some non-coding RNAs [94]. If normal actin dynamics are disrupted, such as under stress conditions, this severely impacts RNA polymerase II activity [70,95]. Similarly, sequestering actin by introducing anti-actin antibodies prevents transcription both *in vitro* and *in vivo*, while actin polymerisation inhibition by latrunculin B also inhibits transcription [88,96]. As actin is also required for transcriptional activity from RNA polymerases I and III, which produce different rRNAs for the ribosome, nuclear actin regulation affects ribosome biogenesis and therefore mRNA translation, in addition to mRNA production by RNA polymerase II [97,98].

Stress causes changes to chromatin structure, transcription factor activity, and transcription of mRNAs and rRNAs. The role of nuclear actin in these changes remains largely uncharacterised, although nuclear monomeric or short polymeric actin structures are sequestered into actin rods [99,100]. This probably depletes the pool of actin available for transcription, limits chromatin rearrangements, and affects transcription factor activity. The dynamic regulation of nuclear actin structures controlling mRNA and ribosome synthesis is pivotal to maintaining protein, and therefore cell, homeostasis in optimal and stressed conditions.

## Actin in RNA export

After mRNA is transcribed, it is processed and assembled into mRNP complexes which are exported to the cytoplasm for translation [101]. Actin is part of at least a subset of these mRNP complexes, colocalising with mRNA export factors in interchromatin granule clusters and DBP40 containing mRNPs [102,103]. Actin is additionally associated with nuclear pore complexes in *Xenopus* oocytes. Sequestering nuclear actin by microinjecting antibodies into *Xenopus* oocyte nuclei prevents export of certain RNAs and RNP complexes [104]. Actin bundles form intranuclear tracks upon which mRNP complexes can be transported, and disruption of these tracks with the actin polymerisation inhibitor latrunculin-B prevents nuclear export of some mRNAs [105]. It is unclear whether this is due to disruption of these tracks, altered mRNP or nuclear pore complex structures, or the prevention of trafficking in the cytoplasm. As nuclear actin structures are altered by stresses, the effects on mRNA export are likely to be relevant to cellular stress responses.

RNA can also be exported out of cells by being packaged into exosomes. These vesicles can either be released into the extracellular milieu or sent directly to recipient cells through tunnelling nanotubes [106,107]. Uptake of these RNA-containing exosomes by other cells can have phenotypic effects [108]. Exosome-derived RNAs are known to be involved in cancer progression and immune regulation [109–111]. Exosome secretion is promoted by RhoA and cortactin, which regulate actin polymerisation [112,113]. Further, exosome internalisation occurs through the actin-dependent processes of phagocytosis and macropinocytosis [113,114]. Delivery of exosomes, whose composition can change depending on the conditions, may play a key role in response to stresses both at the level of the immune system, and individual cells [115,116]. Actin therefore has important roles in both cellular import and export of RNA.

## Actin in mRNA transport and localisation

In the cytoplasm, some mRNAs are transported in translationally repressed particles to particular subcellular locations, where the translational repression is lifted and the mRNA locally translated [117–119]. There are multiple benefits to this phenomenon, including: (1) it is more efficient to transport a single mRNA than several proteins, (2) it facilitates co-translational assembly by bringing mRNAs of a given complex into close proximity with each other prior to starting translation, (3) transporting mRNA to areas with more translation machinery increases the rate of translation, and (4) non-functional (and potentially cytotoxic) interactions are reduced [120–123].

**Figure 4:**
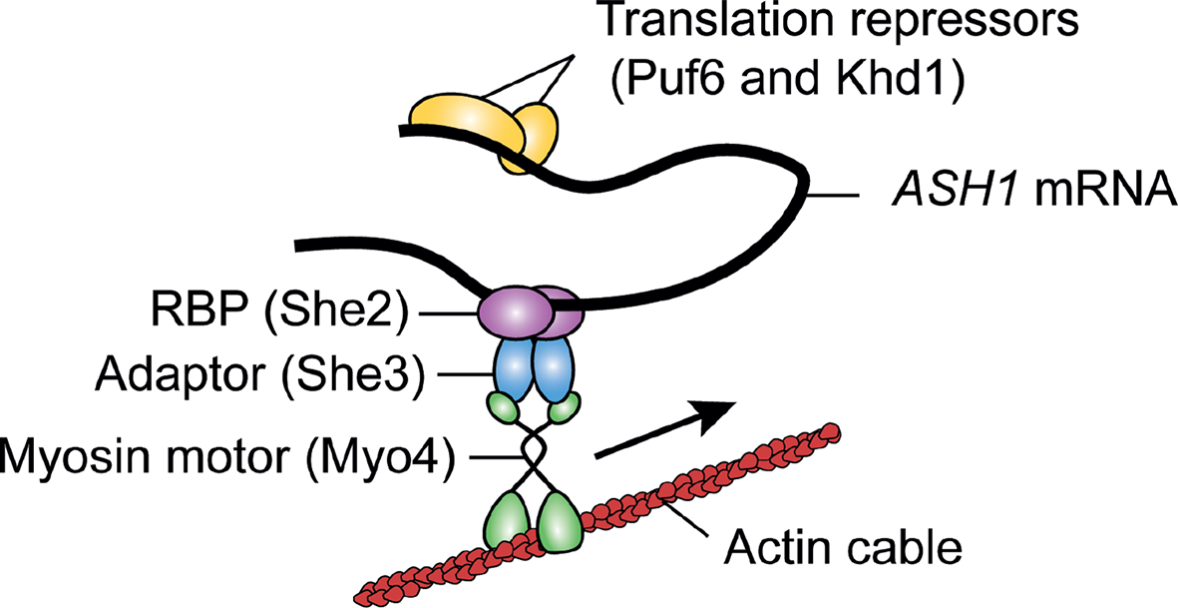
Actin in mRNA transport Some mRNAs, including that of *ASH1*, are moved along actin cables by myosins in budding yeast. mRNAs are bound by the She complex, and maintained in a translationally repressed state by translational repressors such as Puf6 and Khd1. *ASH1* mRNA is transported to the daughter cell (bud), where the translational repression is lifted.

To move to the desired area of the cell, an mRNA which contains RNA localisation elements (also known as RNA zip code targeting motifs) is transported along cytoskeletal structures [124,125]. These motifs, which range from short sequences to multi-partite sequences which coalesce into specific tertiary structures, provide an interface for a *trans*-acting protein(s) to bind. These proteins then facilitate transportation of the mRNA to the desired location [125]. In animals, transport is primarily along microtubules, while for yeast, which have a less pervasive microtubule network, actin cables are used extensively for mRNA transport [15,126,127]. The role of actin in mRNA transport within yeast has been described in detail through investigation of the *ASH1* mRNA. Ash1 protein inhibits mating type switching following mitosis and is localised specifically in the bud/daughter cell [14]. *ASH1* mRNA is transported in a translationally repressed state along actin cables to the bud as part of a complex including the She2 and She3 RNA binding proteins (Figure 4), along with numerous other asymmetrically localised mRNAs including Clb2, Tcb2/3, IST2 and various translation factors [14,15,117]. We have recently shown that the mRNA for the yeast proteasome assembly chaperone Adc17 also appears to be transported along actin cables. This transport moves the mRNA to and from cortical actin patches (CAPs), where it accumulates following stress to help boost translation [12].

Following mRNA transport to the correct region of the cell, its location needs to be constrained. Actin has a major role in retaining mRNA at the cell periphery in both yeast and animals. Tethering of yeast *ASH1* mRNA in the bud is dependent on the presence of the formin Bni1, which produces straight filaments of actin [14]. Actin filaments are also important for anchoring mRNA to the cortex in *Xenopus* and *Drosophila* oocytes [128,129]. Focal adhesions (FAs) in animals and CAPs in yeast are analogous highly branched actin structures located at the cell periphery. FAs and CAPs both interact with mRNAs, notably mRNAs of proteins present in these structures such as β-actin and Las17 (WASP in mammalian cells) respectively [16,130]. The interaction of β-actin mRNAs with FAs, along with the local translation of β-actin, affects FA dynamics. Tethering of the mRNA to FAs significantly increases FA size, which could in turn affect translation of other mRNAs, or activity of FA components which are present at these structures, by providing a larger area for mRNA, protein, and ribosome recruitment and activation [16]. Similarly, Arp2/3 complex mRNAs are localised to protrusions in fibroblasts, and this is impaired when actin filaments are disrupted [131]. Several yeast CAP components have additionally been implicated as RNA binding proteins, suggesting the known mRNAs could just be the tip of the iceberg [132].

Actin-regulated transport and anchoring of mRNA is sensitive to the regulation of actin structures within the cell. Stresses which affect actin organisation in yeast frequently cause mRNAs, including to some extent *ASH1*, to be sequestered in translationally repressed P-bodies or stress granules [133,134]. This is discussed in more detail in the ‘actin in mRNA translation suppression and degradation’ section. This highlights that the role of actin in mRNA transport and localisation is responsive to stress-induced changes of the cell actin architecture.

## Actin in mRNA translation

Once an mRNA has been synthesised and correctly localised, it must be translated to produce a polypeptide, which can be folded into the final protein. Many mRNAs are either known to, or are likely to be, translated at densely branched actin CAP structures (in yeast) and FAs (in mammals) [12,16,69,130,135]. EM studies have shown ribosomes associate with cytoskeletal filaments, while pharmacological disruption of actin increases polysome extraction, collectively suggesting some polysomes interact with actin structures [136,137]. These data, to our knowledge, have not been repeated with more modern techniques and methodologies, however. Together, these data suggest that branched actin structures might provide a platform for mRNA translation. This is supported by two observations: 1) increased translation when *ADC17* mRNA is recruited to CAPs in yeast and 2) that ribosomes are enriched at focal adhesions, where mRNAs are known to be translated in mammals [12,138]. How mRNAs are recruited to CAPs and FAs to be translated is less understood, but it is likely to involve RNA-binding proteins recruited at these locations. Dissection of these mechanisms will be of great interest going forward.

Ribosomes in yeast are distributed throughout the cytoplasm with a significant proportion being membrane-embedded. Ribosomes associate with the endoplasmic reticulum (ER), a proportion of which is associated with the plasma membrane, with ribosomes solely on the cytoplasmic side of the ER [139]. Phase-separated Ede1 structures, which are CAP pre-cursors, are surrounded by plasma-membrane associated ER containing membrane-associated ribosomes [140]. This suggests ribosomes likely additionally associate with CAPs. Further components of the translation machinery which interact with the actin cytoskeleton are superbly summarised in [141]. It is notable that eukaryotic elongation factor 1A (eEF1A) moonlights as an actin regulator, promoting actin polymerisation, bundling, and stress fibre formation [142,143]. Disruption of F-actin decreases mRNA translation, indicating that, as a collective, the above detailed interactions are important [144]. Altogether, these results implicate densely branched F-actin structures such as FAs and CAPs as translation platforms.

FA and CAP distribution and dynamics are sensitive to changes in external cues, including cell stresses, growth factor stimulation, and chemokine abundance [54,56,134,145–148]. Changes in organisation of these structures probably impacts their ability to operate as translation platforms. Altered organisation of actin structures could therefore be a conserved driver of reduced translation upon stress, although proving this for more than a few selected mRNAs remains challenging. One set of mRNAs, encoding the proteasome regulatory particle assembly chaperones, interact more with CAP structures following stress, thereby increasing their translation [12]. These mRNAs are recruited to CAPs following rapamycin treatment by the CAP protein Ede1. Whether these mRNAs directly interact with Ede1 remains to be determined. Interaction of both stress-regulated and non-stress regulated mRNAs with branched actin structures likely dynamically fine-tunes their translation according to the conditions.

## Actin in mRNA translation suppression and degradation

Translation of mRNAs is partly regulated by their incorporation into P-bodies (PBs) and stress granules (SGs). PBs and SGs are structures composed of protein and RNA which suppress translation and, in the case of PBs, mediate degradation [149]. These structures are dynamically regulated in response to the cell environment, in some cases being induced by stresses which also cause significant shifts in cell actin configuration [133,134,148,150]. Supporting a link between actin disruption and PB/SG formation, pharmacological disruption of actin structures affects stress-induced SG assembly. The effect observed varies depending on the actin polymerisation inhibiting drug used, however: cytochalasin B enhances, while latrunculin B inhibits SG formation [151,152]. This difference is likely linked to mode of action: latrunculin B sequesters actin monomers while cytochalasin B inhibits filament elongation and interaction with other filaments [153,154]. Furthermore, profilin, a G-actin binding protein that regulates actin dynamics, is involved in the promotion of SG formation, with many point mutations affecting SG assembly [155]. Finally, PB transportation in plants requires actin filaments [156]. It remains to be seen if this is conserved in other eukaryotes. Interestingly, mRNA localisation to these structures has recently been shown to be compatible with mRNA translation, indicating that they could, in addition to repressing overall translation, contribute toward the translation of specific mRNAs [157]. More research is required to fully understand the functions of actin in PB and SG regulation.

mRNA translational silencing and decay mediated by microRNAs (miRNAs) is an alternative, or complementary, mRNA regulatory mechanism [158]. miRNAs are generally transcribed by either RNA polymerase II or III, giving actin a role in their transcription due to its importance for the function of these polymerases [88,90,159,160]. Several miRNAs have been shown to affect actin-binding protein expression, which in turn can affect the cellular actin structures produced [161–163]. miRNAs, like mRNAs, can be exported to recipient cells where they can affect cell phenotype. This export can be through extracellular vesicles or tunnelling nanotubes [164–166]. Intriguingly, miRNA export can be dependent on the cells environmental context, suggesting stress can contribute to these effects [167].

## Actin in protein activity regulation

**Figure 5:**
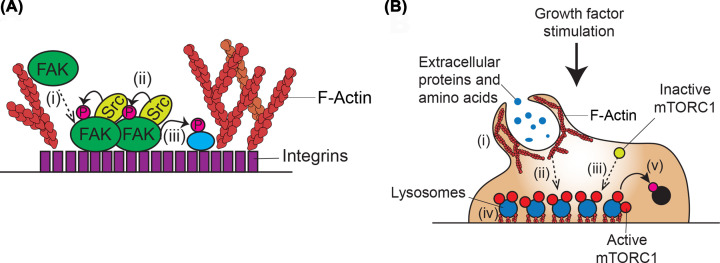
Protein activity is regulated by actin at multiple levels Protein activity depends on its localisation, post-translational modifications, and interaction with other proteins. Actin impinges on one or several of these aspects for multiple proteins, which can in turn affect actin dynamics. (**A**) Focal adhesion kinase (FAK) regulation by and of actin structures. (i) FAK is recruited to focal adhesions (FAs). (ii) At FAs, FAK undergoes a conformational change, oligomerisation, autophosphorylation, and phosphorylation by Src kinase to activate it. (iii) Activated FAK phosphorylates proximal substrates and mediates FA dynamics. (**B**) mTORC1 regulation by and of actin structures. (i) Growth factor stimulation induces macropinocytosis. (ii) Macropinocytosis delivers extracellular amino acids to lysosomes. (iii) mTORC1 is recruited and activated at amino acid containing lysosomes. (iv) These lysosomes interact with FAs. (v) mTORC1 phosphorylates proximal substrates and regulates actin dependent processes including cell motility, autophagy, and cell proliferation.

The activity of a protein is governed by a combination of its localisation, modifications, and interaction with other proteins, including actin. Activity of a protein could be dependent on it localising to a particular region of the cell where specific post-translational modifications are made, which allow interaction with other proteins at that same location. Disruption of any part of this network can result in apparent defects in protein activity. Illustrating this complexity, and the role of actin structures therein, the intracellular protein tyrosine kinase, Focal Adhesion Kinase (FAK), is recruited to focal adhesions (FAs) by binding to nascent adhesion components. At FAs FAK undergoes a conformational change to allow oligomerisation, autophosphorylation, and phosphorylation by Src to induce activation [168–171]. Once active, FAK phosphorylates substrates at FAs including paxillin to regulate FA dynamics (Figure 5A) [172,173]. A further well characterised example is the recruitment of the growth promoting kinase mTORC1 to lysosomal membranes, which is necessary for its canonical activation. mTORC1 recruitment to lysosomes is modulated by lysosomal amino acid content, which increases following growth-factor induced, actin-dependent, macropinocytosis [77]. Following growth factor stimulation, mTORC1-associated lysosomes become more associated with FAs, allowing increased phosphorylation of proximal substrates including S6Kinase (Figure 5B) [174]. In this way, actin structures may act as signalling platforms, although this concept will require further validation. Overall, actin regulates the localisation, modifications, and interactions of several proteins, thereby having a major impact on their activity, and hence protein homeostasis.

As noted in ‘actin in translation regulation’, evidence suggests that actin may provide a platform to concentrate components involved in translation, thus increasing the local translational efficiency. Actin could similarly function as a platform for other multi-step cellular processes. This has been best studied in the context of glycolysis, where many glycolytic enzymes can interact with actin [175–177]. A possible effect of this is to increase the proximity of glycolytic enzymes, thereby limiting the diffusion of each glycolytic intermediate molecule, boosting efficiency of the process [176]. Interaction with actin has opposing effects on the activity of several glycolytic enzymes, allowing opposing regulation of several steps in glycolysis. In contrast with yeast aldolase, mammalian aldolase A is inhibited when in contact with actin, becoming activated when released following insulin stimulation [176,178]. These experiments leave open the possibility that the active fraction of aldolase A may be interacting with smaller actin structures, or still interacting with actin but more weakly. F-actin inhibits lactate dehydrogenase activity *in vitro*, although the *in vivo* effects are not clear [179]. Other *in vitro* experiments have shown that the enzymatic activity of phosphofructokinase and glyceraldehyde-3-phosphate dehydrogenase increases when the interaction with actin is increased [179,180]. *In vivo* validation will be important to understand whether this regulation is physiologically relevant. Structural work will be required to identify the conformational changes underpinning the activation status regulation of these enzymes, although previous work suggests that aldolase may form aggregates on F-actin filaments, potentially promoting actin bundling [181]. Disentangling activity regulation and enzyme concentration regulation to investigate whether actin structures are used as a platform for glycolysis will be challenging.

Actin impacts the localisation of certain proteins through its effects on mRNA localisation (see ‘Actin in mRNA transport and localisation’ section). Actin additionally functions in trafficking proteins and glycoproteins to and from the cell surface in and on endosomes, including protein recycling from the surface of phagosomes and macropinosomes [182,183]. Actin also controls the transfer of proteins between cells, including prions, through tunnelling nanotubes [184,185]. Further regulation of protein localisation is observed in mitotic yeast cells, where actin cables contribute to the asymmetric segregation of protein aggregates into the mother cell, away from the daughter cell [186].

Post-translational modification of proteins is a tightly regulated and highly effective way to fine-tune protein activity through affecting protein conformation and interaction with other proteins. Actin is commonly regulated by proteins whose activity is controlled at the post-translational level, including kinases, G-proteins, deacetylases, and many further modifying enzymes [187–193]. This regulation, which is often responsive to the cellular environment, affects the type, and kinetics, of actin structures which are formed. Several kinases, which regulate other proteins through phosphorylation, including FAK and mTORC1, can be regulated partially by their association with actin structures, including FAs. This binding is important for regulation of kinase activity and varies depending on the environmental regulation of actin structures [77,194,195].

In addition to the role of actin structures in regulating kinases, interactions of actin with other proteins contributes to their activity. In the case of RNA polymerases I, II, and III, interaction with actin is required for their activity (discussed in the ‘Actin in RNA synthesis’ section), while actin nucleators rely on the availability of actin monomers to produce new strands of F-actin. Thick F-actin structures at the cell cortex, which are regulated by the stiffness of the extracellular environment, sequester the E3 ligase TRIM21 and thus regulate substrate ubiquitination in response to external cues [196]. G-actin also provides substrate-specificity for dephosphorylation of eukaryotic initiation factor 2α by the PP1 holoenzyme, allowing restoration of protein synthesis following ER stress [197]. Although not part of normal protein homeostasis, it is notable that the nucelotidyl cyclase bacterial toxins, which are injected into host cells, are substantially activated following their binding to actin, thus restricting their activity to host cells [198,199]. Interestingly, one of these toxins, ExoY from *Pseudomonas aeruginosa*, seems to boost cellular F-actin content, suggesting a positive regulatory loop [199].

Environmentally regulated changes to actin structures impact on protein activity. Multiple stresses (including TORC1 – the yeast equivalent of mTORC1 – inhibition, heat, osmotic, and oxidative stresses) induce depolarisation of the actin cytoskeleton in yeast, redistributing CAPs throughout the cell and weakening the actin cables [54,134,148,200]. Actin depolarisation inhibits signalling by TORC1 likely through activation of Rho1 in the cell wall integrity pathway [201,202]. Actin depolarisation thus affects signalling of TORC1 downstream kinases such as the MAPK (mitogen-activated protein kinase) Mpk1 and the AGC kinase Sch9 [12,203,204]. Conversely, growth factor stimulation leads several mammalian cell types to form large actin-driven ruffles at the cell membrane [77]. These ruffles close to form macropinosomes, which may function both as signalling platforms to boost mTORC1 activity and facilitate nutrient acquisition [77,205]. Actin structures thereby affect mTORC1 signalling, while the reverse is also true with mTORC1 affecting cell migration, the formation of actin-containing autophagic structures, and cell cycle progression, ultimately leading to cytokinetic ring formation [206–208]. Actin structures, and their dynamic responses to the extracellular environment, thus affect the activity of many proteins in a myriad of ways.

## Actin in protein degradation

Degradation of damaged or short-lived proteins (e.g. cell cycle proteins or stress response proteins following stress relief) is key to maintaining protein homeostasis and commonly deteriorates with age [209]. The importance of protein degradation is particularly apparent following cell stress when many proteins are either damaged or need to be degraded to allow proteome adaptation. Two major mechanisms exist for protein degradation: the ubiquitin–proteasome system (UPS) and macroautophagy (hereafter autophagy), both of which are upregulated upon stress [210,211]. The UPS is responsible for degradation of most short-lived proteins, while autophagy degrades most long-lived proteins and larger material, such as organelles and protein complexes, including the proteasome [212].

The proteasome is a multi-subunit protein complex composed of a core particle (CP) and one or two regulatory particles (RPs). Both the CP and RP are dependent upon assembly chaperones for efficient assembly [23]. In yeast, when actin is perturbed, RP assembly chaperone (RPAC) mRNA interacts more with densely branched CAPs to facilitate increased translation and hence promote proteasome assembly [12]. It is not known whether mRNA interaction with CAPs is required for basal RPAC protein expression, but this interaction could be important under non-stressed conditions as well. Yeast proteasomes interact with actin filaments, although the inhibition of actin polymerisation does not appear to diminish proteasome activity [12,213]. Proteasome CPs have further been shown to interact with actin in fibroblasts [214]. In neurons, following stimulation, the proteasome is localised to and retained in spines by association with actin, and increased activity is observed [215]. Proteasomes are sequestered at actin aggregates formed by administration of the actin stabilising compound jasplakinolide [216]. Jasplankinolide treatment reduces proteasome activity in cell lysates, although this is probably due to sequestration of proteasomes at insoluble aggregates rather than a direct effect on proteasome activity. Finally, proteasome CPs have been found in exosomes, export of which is an actin-dependent process, and may contribute to the immune response [217–219]. Alternatively, it may be the case that exosomal CPs are due to faulty exosome loading. The mechanistic basis of CP loading into exosomes remains to be identified. Actin structures and dynamics impact proteasome activity on a whole cell, and potentially whole organism level, by facilitating the exchange of cellular contents through exosome secretion and uptake, although whether this is the case remains to be seen.

Autophagic degradation of proteins involves the formation of a cup-shaped membrane structure (phagophore), which encircles the material to be degraded to form a double-membrane vesicle (autophagosome). The autophagosome subsequently fuses with lysosomes, and the engulfed material is degraded [220]. Although the material degraded by autophagy isn't limited to proteins, organelles such as mitochondria are replete with numerous proteins. Actin has roles throughout autophagy and is assembled in an autophagy-dependent manner at protein aggregates [187,221]. Branched actin is assembled at early omegasomes, a precursor to the phagophore, by Arp2/3 complex activation in the initial stages of starvation-induced autophagy. When actin assembly is impaired, both using pharmacological means and mutation of actin regulatory proteins, autophagy initiation (but not maturation) is impaired, while increasing actin assembly increases autophagy [222,223]. Actin structures at the omegasome are thought to provide structural support to the phagophore, which expands around them [224]. Phagophore expansion requires delivery of Atg9a containing vesicles from the Golgi to supply membrane, a process dependent upon Arp2/3 actin nucleation activity regulated by Annexin 2 and WASH [225–227]. Following phagophore closure and transport of the autophagosome to the lysosome, autophagosome–lysosome fusion occurs. Actin remodelling by recruitment of cortactin, and subsequent Arp2/3 activation, is required for autophagosome fusion with lysosomes during selective autophagy of protein aggregates but not starvation-induced autophagy [187].

Proteasome activity can be important for actin structure regulation. In neurons and B cells, proteasome activity is important for the dynamic regulation of actin structures, although this is not conserved between all cells and conditions [228–230]. Conditional proteasome mutants in yeast additionally have altered actin morphology [213]. Autophagy is important for changes to actin orientation induced by mechanical stress [230]. Protein degradative activity by the UPS or autophagy and actin organisation regulation therefore goes both ways.

## Summary

Actin is fundamentally important to the proper functioning of many cellular processes. It is becoming increasingly clear that protein homeostasis maintenance is one of these processes. As described here, the dynamic regulation of actin structures is key to maintaining protein, and thus cell, homeostasis in response to changing environmental conditions. Actin is involved in regulating mRNA and protein abundance at multiple stages from synthesis to degradation. Actin, as one of the most abundant and dynamic molecules within the cell, which can be rapidly reconfigured into multiple structures, is an ideal protein to tightly regulate changes in protein production to account for the cell environment.

## Future directions

As might be expected with such a broad topic, there are many avenues which still require further research. Actin interactions with the translation machinery have been extensively identified, but the challenge remains to associate those interactions with specific actin structures and to identify whether those interactions are direct or indirect. It is likely that the recruitment of a specific mRNA to a specific actin structure will often require one or more intermediate RNA-binding proteins. Additionally, mRNAs which interact with and are regulated by actin require systematic identification and characterisation. The binding sequences within mRNAs important for recruitment to actin structures, along with proteins directly bound to the mRNA, will also be important to identify. A major open question is whether and how the actin cytoskeleton interacts with P-bodies and stress granules, and the reasons underpinning different effects on their structure by different actin-disrupting small molecules. Characterisation of nuclear actin structures and regulation remains a formidable challenge. Whether different actin isoforms differently contribute to these processes is also a topic of interest. While technically demanding, exciting discoveries regarding the role of actin in protein homeostasis maintenance upon stress will lead to fundamental improvements in knowledge of the systems underpinning protein and cell homeostasis maintenance, a critical requirement for all living things.

## Open Access

Open access for this article was enabled by the participation of University of Dundee in an all-inclusive *Read & Publish* agreement with Portland Press and the Biochemical Society under a transformative agreement with JISC.

## Abbreviations

ABP: actin-binding protein
CAP: cortical actin patch
CP: core particle
FA: focal adhesion
PB: P-body
RP: regulatory particle
RPAC: RP assembly chaperone
SG: stress granule
